# Cereal aphid movement: general principles and simulation modelling

**DOI:** 10.1186/2051-3933-1-14

**Published:** 2013-12-23

**Authors:** Hazel R Parry

**Affiliations:** CSIRO Ecosystem Sciences, GPO Box 2583, Brisbane, QLD 4001 Australia

**Keywords:** Long-distance movement, Migration, Cereal aphid, Flight, Simulation modelling

## Abstract

Cereal aphids continue to be an important agricultural pest, with complex lifecycle and dispersal behaviours. Spatially-explicit models that are able to simulate flight initiation, movement direction, distance and timing of arrival of key aphid species can be highly valuable to area-wide pest management programmes. Here I present an overview of how knowledge about cereal aphid flight and migration can be utilized by mechanistic simulation models. This article identifies specific gaps in knowledge for researchers who may wish to further scientific understanding of aphid flight behaviour, whilst at the same time provides a synopsis of the knowledge requirements for a mechanistic approach applicable to the simulation of a wide range of insect species.

Although they are one of the most comprehensively studied insect groups in entomology, it is only recently that our understanding of cereal aphid flight and migration has been translated effectively into spatially-explicit simulation models. There are now a multitude of examples available in the literature for modelling methods that address each of the four phases of the aerial transportation process (uplift, transport in the atmosphere, initial distribution, and subsequent movement). I believe it should now be possible to draw together this knowledgebase and the range of modelling methods available to simulate the entire process: integrating mechanistic simulations that estimate the initiation of migration events, with the large scale migration modelling of cereal aphids and their subsequent local movement.

**Electronic supplementary material:**

The online version of this article (doi:10.1186/2051-3933-1-14) contains supplementary material, which is available to authorized users.

## Introduction

Although there are approximately 5,000 species of aphid across the world, only a handful pose a threat to cereal production. Those that do can commonly be termed ‘cereal aphids’, which can cause both direct damage to cereal crops such as wheat and barley but importantly transmit a number of viruses, such as Yellow Dwarf Viruses, that can cause even greater damage to crops. The most common cereal aphid species globally are the Rose-Grain aphid (*Metopolophium dirhodum* (Walker))*,* the Grain aphid (*Sitobion avenae* (Fabricius)), the Bird Cherry-Oat aphid (*Rhopalosiphum padi* (L.)), the Corn aphid (*Rhopalosiphum maidis* (Fitch)) the Russian wheat aphid (*Diuraphis noxia* (Kudjumov)), the Indian Grain aphid (*Sitobion miscanthi* (Takahashi)), the Rice root aphid (*Rhopalosiphum rufiabdominalis* (Sasaki)), the Apple grass aphid (*Rhopalosiphum insertum* (Walker)), the Blackberry-cereal aphid (*Sitobion fragariae* (Walker)), the Fescue (or grass) aphid (*Metopolophium festucae* (Theobald)), and the Greenbug (*Schizaphis graminum* (Rondani))*.* For a comprehensive account of all aphids found on cereal crops please see Blackman and Eastop [1]. Common to this set of highly successful r-strategist aphid species are frequent movement and a propensity to migrate over long distances, thus having a high disease vectoring capacity to cause widespread damage to cereal crops. ‘Migration’ is taken to be the periodic flight of insects beyond the boundaries of their old breeding habitats into new ones, where migrant behaviour refers to individuals “that are relatively undistracted during flight by stimuli that normally lead fairly quickly to the satisfaction of normal appetites and especially to oviposition” [2] pp 19. This definition is taken to apply equally to non-ovipositing morphs which form a significant proportion of aphids that migrate to commercial crops.

Both an understanding of, and ultimately the capability to predict, aphid movement patterns at multiple spatial scales are vital to achieving area-wide strategies for integrated pest management of cereal aphids [3]. Area-wide pest management programmes need to incorporate approaches that can forecast the timing and magnitude of pest immigration events taking into account potential source populations. This would allow for a regional approach to pest management, thinking beyond the crop rather than employing simply a reactive within-field response. To this end, spatially-explicit models that are able to simulate flight initiation, movement direction, distance and timing of arrival of key aphid species can be very valuable [3].

Many observational studies have shown that aphids are capable of very long distance movement. A number of studies of aphids in the USA and inferences on their migration pathways in relation to jet-streams are summarised in Wallin and Loonan [4]. Field observations in conjunction with a study of the timing of low-level jet winds confirmed that aphids were efficiently transported by jet winds from the Southern Plains to the North-central states of Iowa and Wisconsin. Since as early as 1925, trans-oceanic migrations of alate aphids have been noted [5]. Studies have also noted evidence of long distance migration between Australia and New Zealand [6]. More recently, evidence of limited genetic variation across large regions points to high levels of long range dispersal activity in cereal aphids, e.g. in Russian wheat aphid in the USA [7] and *S. avenae* in Britain [8].

Several comprehensive reviews exist on the ecology of aphid flight [3, 9–14]. Much of the research conducted in the 1950s to 1960s was founded on the theory that the flight of alate (winged) aphids can be separated into two phases. The first is a distinct migratory phase, followed by an ‘appetitive’ (foraging or mating) phase [2, 15, 16]. Some attributed the two phases to the weather conditions, with predominantly passive transport in windy weather and active flight in calm weather [17–19]. However, more recent authors see this more as a continuum [20], where the transition between the two can be influenced by a wide range of factors [21].

The early studies also maintained that most flights are migratory, with only a small proportion of flights being from plant-plant [15, 19]. More recent work declares that this interpretation of ‘migration’ is ‘overstated’ [14]. Recent literature now agrees that alate aphids tend to move mainly short distances over their lifetime, of the order of 20 m in favourable habitat and 100 m in poor habitat: “spatial displacements (dispersal) rather than migration *sensu stricto*, are the ‘norm’” [20], pp 1479, [22]. Migration is an infrequent occurrence, the ‘exception rather than the rule’ [14], pp 293 and there is a gradation from ‘non-flyers’ to local flyers and then migrants. However, although migration may now be considered the exception across all alates, newly emerged alates are highly likely to attempt migration at the earliest opportunity, although this window of opportunity is small (further details in section Basic rules of aphid flight). Subsequently, if an aphid encounters an unsuitable host it will move on, trying to maximise the chances of finding a suitable host in the shortest possible time [14]. There is a large degree of stochasticity in flight behaviour, and even a large variation in duration of migratory behaviour between clonal individuals [23, 24]. Overall, aphid flight activity is now best viewed as a complex continuum of behaviour at multiple spatial and temporal scales of dispersal from the plant scale to global, with a large range of possible flight activity spanning both inadvertent and intentional flight [3].

“Many researchers have confused host-alternation and migration and incorrectly referred to both as migration. The two terms are not synonymous and they describe separate behavioural phenomena” [9], pp 462. This paper concentrates on the primarily temperature- , crowd- and long day- induced winged females (summer alate virginoparae) which fly to fresh summer hosts or migrate between crops and grasslands as anholocyclic populations. This is opposed to the short-day induced, winged autumn forms that return to the primary (non-crop) host (gynoparae). It is known that there are distinct behavioural differences between gynoparae and virginoparae [25–28], such as a faster initial climb rate and longer flight duration in gynoparae. Together the result is that where holocyclic and anholocyclic populations combine in cropping regions such as France, they tend to exhibit bimodal annual migratory behaviour [9, 29, 30].

**Figure 1:**
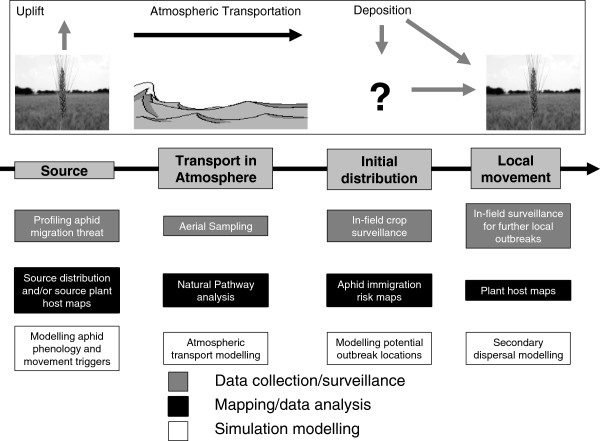
**Key phases in the aerial transport process for cereal aphids and the relationship with data collection, risk mapping/data analysis and simulation modelling objectives.**

In this paper I firstly outline the four key phases in the aerial transport process applicable to aphids, as defined by previous authors. I then describe four core ‘basic rules’ of aphid flight that I perceive to be of key importance, which provide a framework for a simulation model and upon which further complexity can be added, as detailed in the following section ‘nuances of aphid flight behaviour’. I then give an overview of simulation models of aphid flight developed to date, showing modelling achievements. This includes a summary of the approaches that have been used, as well as examples of data collection methods and surveillance that can help inform such models. This indicates where more can be done to integrate approaches in order to simulate the entire aerial transport process (Figure 1).

## Review

### Basic rules of aphid flight

There are four key phases in the aerial transport process for cereal aphids (Figure 1): (1) uplift at the source, (2) transportation in the atmosphere, (3) deposition leading to the initial distribution following transportation and (4) subsequent local movement, after [31, 32]. In relation to each of these four stages, scientists collect various forms of data and conduct surveillance, map and analyse distributions and also construct simulation models, as indicated in Figure 1. By thinking about the aerial transport process in this way it is easier to assess the overall principles of aphid migration and also to consider where knowledge gaps exist, as well as how we can bring together understanding from each of these four phases to better simulate the aerial transport process [32].

**Figure 2:**
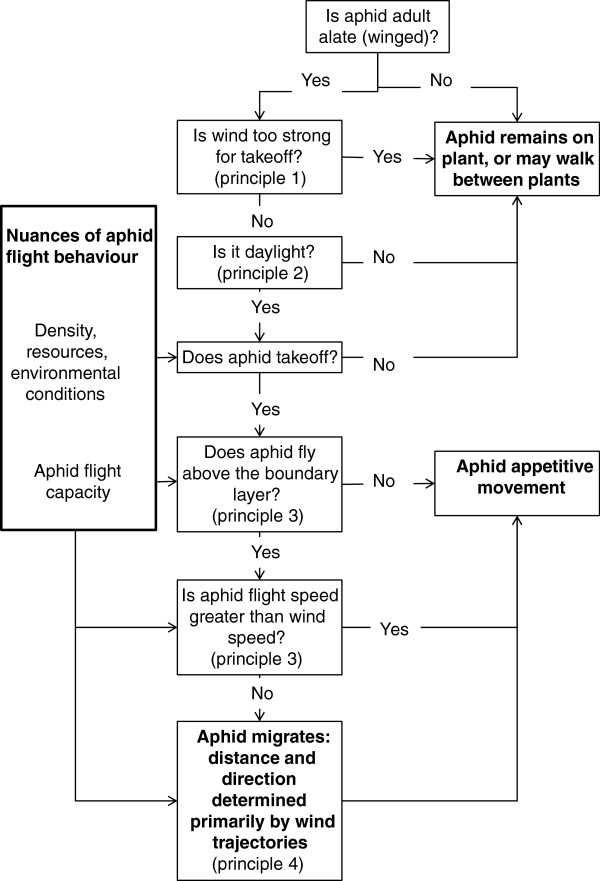
**Conceptual model of cereal aphid flight.**

Although aphid flight is a complex phenomenon acting at multiple spatial and temporal scales [3], in order to reach a general understanding of aphid flight and move towards predictive mechanistic simulation of this process, a general set of quantifiable rules must be formulated as a basis on which we can build. To this end, I propose that the rules of migratory flight of cereal aphids (that may also apply to a wide range of other aphids) can be conceptualised as following four general principles, which are summarised diagrammatically in Figure 2 and the numeric parameters given in the text are summarised in the Additional file 1 as a quick-reference table.

Firstly, the primary factor influencing the initiation, path, speed, distance and duration of aphid flight is wind speed and direction. The majority of studies of these wind effects are conducted in the laboratory, and although they yield quantitative values some consideration should be made of their relevance in the field and thus how these parameters should be best applied in a model. For example, Walters and Dixon [33] recognize that winds are ‘gusty’ in the natural environment, and therefore even when mean wind speeds are high there are likely to be periods where the wind speed lowers and allows for take-off. There is strong agreement in the literature that once aphids go above a ‘boundary layer’ at approximately 1 m from the ground aphid movement is controlled by the wind [34–37]. Flight below this boundary layer can be termed voluntary ‘appetitive flight’. Aphids undertake appetitive flight if wind speed is not above 8 kmh^-1^[15, 38–44], with their flight path influenced by wind speed as low as 2 kmh^-1^[14]. Although it should be noted that there is some evidence that if wind speeds are higher than 8 kmh^-1^ aphids will migrate, but with a delay ([38]; for a graph and equation relating flight occurrence and wind speed see [33]). Aphids will still take off in wind velocities of approx 10–11 kmh^-1^[38]. Continuous wind velocities of 4.8 kmh^-1^ caused a delay of 4–10 hrs, and wind speeds of 8 kmh^-1^ caused a delay of 24 hours or more for summer migrants of *A. fabae* and *B. brassicae*, however, after these extensions migratory flight occurred with regularity [38]. This probably accounts for evidence from field studies of aphids flying in much higher winds [19].

Second, aphid migration will take place within a day (24 hours) and primarily during daylight; aphids have a strong preference for daylight flight initiation [45, 46] with take-off increasing almost linearly as a function of light intensity in *S. avenae* and *R. padi*[47]. Field studies have shown take-off occurred at light intensities of 1000 lux (approx 3.85 Wm^-2^) and higher, with no upper limit [12]. Cool air at night in temperate climes means that the upper air clears of aphids at night [12, 14, 39, 48, 49]. There is usually a double peak of migrants in the day, the first peak being the morning migration when light and temperature allow, the second being alates that mature during the day [19, 30, 39, 48]. Wiktelius’ [48] study shows that although flights are bimodal in early and mid-season (spring and summer in Sweden) the diurnal flight periodicity is unimodal during autumn migration, due to shorter days and lower temperatures. There is also some evidence flight can continue overnight as aphids become entrained in high altitude atmospheric layers (particularly with warmer temperatures, such as experienced in Australia and the USA [6, 13, 50, 51]), thus increasing the window of aphid flight making movement more difficult to forecast in such regions [32]. Migrating over-night as well as during the day means aphids may displace further, as the potential scale of displacement is greater [50, 52–54].

Thirdly, an individual can only migrate a distance of several kilometres once (if at all) during its lifetime [22, 50, 55], and that single long distance migratory flight is most likely to be the aphid’s maiden flight [3, 12]. There is evidence that many species show wing muscle autolysis in as little as 2–3 days after moult [14, 28, 46, 56–60]. There is also known to be a ‘waiting period’ also termed the ‘teneral period’: the period between moulting and taking flight, which varies from 6–36 hours [19]. Due to this as well as environmental limitations to flight, it is considered that in fact aphids spend much of their lives unable to fly [3], with migratory flight only possible via active take-off and ascent within a limited time window of approximately four days from moulting [56, 61, 62]. The aphid’s take-off angle will also become more acute during this time, limiting the potential to reach the planetary boundary layer and thus travel long distances [52, 63]. However, this doesn’t mean that aphid migration is a rare event; it is common, although seasonal [3], and it is likely that all alate aphids will attempt to migrate during their lifetime at least once given the opportunity: which will be constrained to a window as defined above.

Finally, ‘normal’ migration will generally last only 1–3 hours [14, 15, 25, 26] and is known to be as short as 30 minutes or less for spring and summer migrants [25, 26]. The percentage of aphids remaining airborne after a given time is also estimated in the literature, where only 0.1% of aphids are estimated to remain airborne after 3 hours [64]. The resultant dispersal distance within this timeframe would be of the magnitude of tens of kilometres from a source, as shown in South Australia for *R. padi* along a transect from an irrigated pasture source through annual grasses [65]. However, this may be an underestimate, as higher mean flight durations to exhaustion for cultured aphids of 5.3 hours and 8.9 hours for field-collected aphids have been observed, with a positive correlation of flight duration with aphid weight [66]. This study aligns with the flight capacity of tethered *R. maidis* studied by Liquido and Irwin [61], who found a strong effect of aphid age and lipid content: alate adults under a day old could fly for up to 14.3 hours, two-day old aphids flew for a maximum of 6.5 hours, and four-day-old aphids flew a maximum of 1.75 hours. During migration the aphid is assumed to be carried by the wind a distance determined by the flight duration multiplied by the wind speed, in the direction of the wind’s movement [14, 38]. However, it is now known that aphids do not behave in an entirely ‘passive’ manner whilst migrating with the wind, particularly in relation to the altitude gained [64] (see In flight, below).

Subsequently, repeated alighting, brief probing and re-take-off from hosts and non-hosts is a common pattern among Aphididae [67, 68], generally termed ‘appetitive flight’.

### Basic rules of appetitive flight

Alate aphids lose control of their flight at wind speeds of around 2 kmh^-1^[14, 38]. Thus it can be inferred that appetitive flight may occur at low wind speeds (2 kmh^-1^ or less), taking the form of increasingly ‘random movement’ as wind speeds lower, and short flights tend to be concentrated around host plants [67]. Harrington *et al.*[69] found that 27% of winged R. padi re-takeoff at least once. Appetitive flight behaviour is thought to differ between alate aphids that have undergone migration and those that have not, as their degree of rejection of alighting surfaces differs [9]. In general, the flight speed of aphids is between 0.8-3.3 kmh^-1^[12]. This is in agreement with Compton [36] who states that the maximum flight speed of aphids is 0.9 ms^-1^ (3.24 kmh^-1^). To obtain the maximum distance flown by foraging aphids, this maximum flight speed can be multiplied by the total foraging flight time of an aphid, which is about 30–240 minutes [15], pp 84. The resultant maximum distance would be around 200 m (without wind assistance). Individual appetitive flights will be much shorter than this, just a few metres [70] where aphids fly for only a few seconds up to around 5 minutes at a time, depending on the suitability of the habitat and aphid exhaustion [15]. Aphid flight capacity for self-propelled flight has also been shown to be strongly influenced by the age of the aphid [61] and also to be influenced by whether the aphid is viruliferous (*R. padi* with BYDV): nonviruliferous aphids over 40 hours old were found to have a greater flight capacity than their viruliferous equivalent [62]. Other factors known to influence aphid appetitive flight include plant structure and crop architecture [71].

Apterous (wingless) aphids move from plant to plant very small distances, at a speed of around 5–20 cm min^-1^ depending on the species [12], or may ‘run’ from 15–35 cm min^-1^[12, 23, 67]. Although small, it is thought that these movements of apterae are significant, allowing aphids to spread locally through crops more efficiently than by flight alone [72, 73], particularly as a response to disturbances such as wind, herbicide, predators, crowding, mechanical disturbance, drought and virus-infected plants [73] (but notably not rain, although others have found rain effects to be important [74]).

Beyond these basic principles, a fully mechanistic understanding of short-range, appetitive dispersal of aphids has not been reached, as there are a multitude of factors that can influence the short-range flight initiation, distance and landing of the aphids that act often simultaneously with varying importance that is not well understood, with often conflicting results reported in the literature [3]. Such factors that may influence the flight of aphids include landscape elements (such as hedgerows and trees), cropping systems and crop phenology [75]. It is possible for these to be taken into consideration in a spatially explicit simulation model e.g. [76].

### Nuances of aphid flight behaviour

Although it is possible to construct some very general rules that might be viewed as broadly governing the aphid movement process as above that can form a basis for a model of aphid flight, it would be a mistake to consider that these rules apply universally to all aphid species and to all alate morphs. This section now addresses some of the complexity of aphid movement, and a range of additional important biological and environmental factors that should also be considered in understanding and simulating aphid migration.

#### Take-off

Aphids are very sensitive to a range of environmental factors that determine whether they take-off. These include crowding, wind speed, temperature, day length, light levels, humidity and crop growth stage or quality. Not all factors always apply, depending on physiological traits of the aphid such as age and morph. For example, gynoparae have a greater tendency to short-day induced migration, however other winged forms are less likely to show migratory urges during maiden flight [14]. Temperature, photoperiod and crowding also determine the proportion of alate morphs forming in the population [20, 77, 78], for example as described for alate viviparous females of *R. padi*[79] and for *S. graminum*[80], where host plant was also found to be a factor. This is alongside genetic factors and is still a large area of ongoing research, for a recent review see [81].

#### Host plant conditions

Aphid starvation is thought to be a significant factor affecting flight initiation [82], as well as host plant quality (including the presence of the virus BYDV) [62, 83] and the presence of natural enemies [6, 44, 84]. Although many of the cereal aphid species feed on multiple grass hosts, periodic harvesting of crop hosts or dieback of grasses during drought may stimulate mass movement. Crop growth stage may instigate migration; alate aphids were stated to disperse from crops (in Victoria, Australia) during the October-November period ‘as the crop matured’ [6]. For some key cereal aphid species such as *R. padi* and *S. avenae* a relationship between host plant quality and dispersal has been observed [77], although this same study shows that across all aphid species there are conflicting results on the relationship between host plant physiology and the propensity for aphid movement.

Plant structure and crop architecture also have an important influence on flight initiation as they affect the ability of aphids to move up above the plant boundary layer and affect local environmental variables such as wind speed to permit take-off [68], with some indication that in mixed cropping aphids depart more readily due to increased wind barriers and shade effects [44].

Crowding may stimulate flight as jostling of aphids on the plants can pre-condition alates for flight take-off [33, 46, 85]. Furthermore, migration has been associated not just with crowding in the adult stage, but also with crowding experienced in nymphal stages leading not only to wing formation in the adult stage but also to increased migratory behaviour [86]. Pseudo-crowding mechanisms are also thought to result from aphid response to the presence of natural enemies, thus flight initiation may be partially attributed to natural enemies in this way [3]. Natural enemies may also simply disturb aphids into moving away and falling off a plant or initiating flight that can lead to both long- and short-distance dispersal [3] and references therein. Overall, the relative importance of natural enemies to dispersal initiation and the interaction with environmental factors, aphid density or plant quality is unclear [3].

#### Environmental factors

Low temperatures have been shown to inhibit the take-off of cereal aphids in the field [33]. Although the threshold for flight of cereal aphids is given as about 14-15°C [45], in reality this threshold is a guide only and not a firm limit. Movements on a daily timescale are adaptable and can simply be delayed by low temperatures and are known to be region-specific see also [48, 75, 87]. For example, *R. padi* adapt to the conditions at the season of their development and any delay experienced due to adverse temperatures lasts only for a matter of hours, and is not believed to actually prevent movement of the alate morphs [33]. Likewise, high temperatures may also inhibit the take-off of cereal aphids, with the maximum temperature threshold presumed to be around 31°C [12], but with some exceptions such as *S. graminum* with a threshold of 41-42°C [12]. However, less is known about how high temperatures may impact on aphids other than on aphid mortality, and more research is required [88].

Early studies indicated that dry conditions favour aphid migration [40]; humidity will inhibit take-off if it is greater than 70% [41, 89, 90], although other studies have shown this effect is so temporary that it can be considered irrelevant [91]. There are also studies that have evidence to the contrary, for example high soil moisture is associated with high aphid populations and thus increased migration [92]. The mechanism of the impact of humidity is unclear; whether directly on flight behaviour or indirectly on reproduction. Therefore this is an area that could benefit from further investigation [19].

#### In flight

Migrant alate aphids ascend rapidly up into the atmosphere if they are carried by convective updrafts with rates of ascent measured at up to 3 ms^-1^[50]. Even without an updraft, aphids may ascend rapidly when in a migratory state, e.g. *Aphis fabae* ascend at 0.25 ms^-1^[93], climbing fastest soon after take-off though the rate may fluctuate [15]. Aphids may reach very high altitudes, including the low-level jet stream [4, 51]. In general, aphids can be carried long distances by winds at 300-1500 m [91]. Below 1 km (in the atmospheric convective boundary layer (CBL)) insects, including aphids, are common in the atmosphere and are generally concentrated in well-defined plumes [94]. Whilst they may even gain heights of around 2.5 km [95], their density generally decreases systematically with altitude [54]. At lower altitudes where aphids are common, topography may have a significant impact on long distance dispersal, including mountain ranges and trees [65]. Temperatures influence the distribution of aphids in the atmosphere, for example temperature inversions leading to atmospheric stratification, which can inhibit upward movement of aphids at times or entrain migrant aphids within the planetary boundary layer [54].

There is evidence that there is a behavioural component to aphid flight even whilst in the airstream, so that aphid flight should probably not be considered entirely ‘passive’ [64]. There is also evidence that rain may increase both the amount of aphid movement and the distance travelled, although rain also leads to increased mortality [4, 92, 96].

Due to the strong influence of wind on aphid flight, long-distance movement of aphids may not actually be due to migration, rather due to accident where the aphid may have been caught in an updraft that has led to inadvertent long-distance wind-borne displacement [12, 97]. However, this is much less common than migratory long-distance movement [3], and so in general it can be assumed that long-distance flights are predominantly due to migratory behaviour in aphids.

#### Landing

Although at what point and for what reason during their flight aphids decide to land remains largely unknown [31], one of the key factors that appears to determine when an aphid lands is renewed visual responsiveness to plant-related wavelengths. Aphids are attracted to wavelengths > 500 mμ, especially yellow, also green and orange [47, 98]. They have been shown to actively bypass blue to ultra violet spectrum when landing (whereas at take-off they are highly responsive to this wavelength) [47]. This has been tested in the field, but some species are less attracted to certain wavelengths than others [12, 14, 36, 98]. This knowledge has translated into integrated pest management strategies in horticultural crops, with the use of silver reflective plastic mulch to deter the arrival of aphid disease vectors [99].

Physical factors such as exhaustion are postulated as possible causes of flight termination; however lab experiments and field studies have not linked lipid reserves with flight distance [31].

3-D visualisations and analysis of aphid flight in the laboratory and the field have been conducted [100], these show that when landing, aphids compensate for wind direction and strength in order to maintain flight path direction and that they land preferentially into the wind. However, even if an aphid switches behaviour to try and land, it may be such that meteorological conditions such as strong airflow inhibit landing, particularly for aphids flying in low-level jet streams during nocturnal hours in warmer climes [54]. Another meteorological factor is precipitation, although it is not clear in the literature the importance of this [31, 75]. Precipitation may wash some aphids from the air [91, 101]. Heavy rain (large raindrops) is probably most effective at cleaning the air, however heavy rain immediately after aphid fallout may even kill newly arrived aphids on the plant [4, 102]. Temperature may also have an influence on whether an aphid continues to fly, and these thresholds differ considerably from take-off temperature thresholds [60, 103].

Landscape structure at multiple spatial scales may be important in determining aphid landing behaviours and arrival rate; however there has been little work done on this to date, with landscape studies mainly focused on abundance of aphids and their natural enemies. There is some within-field evidence that mixed cropping reduced landing rates of aphid species on sorghum and soybean, compared to soybean monocultures [104]. Crop variety and configuration, as well as growth stage, may therefore have an important influence on aphid landing [83]. Some studies indicate that host density is not important in determining whether an aphid lands [12], although there is some evidence to the contrary where ground cover appears to be important [104].

Overall, the termination of aphid flight remains a particularly complex area where less data is available and fewer conclusions have been reached to date. As Hendrie *et al.* state, “We initially theorized that the factors determining flight termination and their interdependence would be much simpler than they proved to be” [31], pp 567.

### Simulating aphid migration

To simulate aphid migration it is necessary to bring together process-based population dynamics models to model flight initiation (Figure 2) and long-distance dispersal simulation techniques using wind trajectories^a^. Such integrated simulation methods are now available to study the entire aerial transportation process through four phases: at the source, in the atmosphere, the initial distribution following transportation and subsequent local movement and risk (see Figure 1). Knowledge of the potential source locations of aphids is therefore highly important along with their population dynamics at that location, but in many cases this may not be known [3]. However, in this case a trajectory modelling approach may also allow for identifying the source of an aphid outbreak using ‘back-trajectories’ e.g. [105].

The concept of trajectory modelling for long distance migration of aphids is not new [31, 106]. However, due to the volume of data required, there are numerous challenges to transferring, storing and processing atmospheric data for trajectory models. Therefore, many models that have explored aphid flight in the past have focused primarily on flight initiation (often data-driven rather than a simulation approach) and local dispersal modelling, rather than long distance migration (with the notable exception of the trajectory simulation models of Isard *et al.*, although these are also data-driven). Recently the entomological data collection tools, meteorological data, modelling techniques, computational power and knowledge about aphid migration behaviour have become sophisticated enough to begin to establish realistic and robust long distance migration simulations. There is also now the opportunity to begin to integrate methods to simulate multiple phases of the atmospheric transportation process (Figure 1). Here I give a summary of existing models of aphid flight and the range of data sources and data collection methodologies, in addition to the rich literature already described, that can help to parameterise and validate them.

#### Data collection

There are a number of observational methodologies and examples of studies that have attempted to track insect movement [11, 14, 107]. These include back-tracing, to find out from where insects may have originated [6, 106], direct tracking techniques such as radio-labelling [108], suction trapping, used widely in the UK and Europe, as well as the western USA [109–114] and aerial sampling [31, 53], including the use of aircraft-mounted sampling devices [54]. Other field-based studies have observed landing rates that are indicative of aerial movement [55], including with the use of pan trapping methods or small-scale suction sampling e.g. [115]. Recently, video and radar observation methods have been applied [116, 117] using concepts developed in the late 1980s to detect aphids by the Pest and Weather project of Illinois [118], along with the use of molecular markers and genetics [8, 109]. Genetic and electrophoretic methods for back-tracking are beginning to help us understand where various airborne aphids came from [119–121]. Combinations of these methods have given valuable insights, such as identifying whether aphids are more likely to be originating from long-distance or local sources [122], using a combination of suction trap, field sampling and genetic data analysis. However, the challenge of obtaining concurrent measurements of profiles of aphid density alongside atmospheric and environmental variables as highlighted by Isard *et al*. [54] largely remains, along with a full understanding of the physiological limits that determine the duration and capacity of aphids for long-distance movement.

#### Existing aphid flight simulation models

Although a range of methods and ongoing surveillance for aphid data collection exist, the spatial coverage is patchy and overall there are limits to what knowledge can be gained by observation alone about aphid flight behaviour, without also exploring with simulation modelling approaches [123]. A recent summary of some existing aphid population dynamics models exists [75]. However, the majority of models summarised in that review are aspatial, focused on the population dynamics of the aphid with simple parameters or equations to represent immigration e.g. [124].

Some models have simulated cereal aphid movement or the timing of arrival of migrants into crops (Table 1). In general, these have done so by either ignoring complex population dynamics of the aphid and focusing on atmospheric processes along with simplified behavioural rules [64] or by coupling with observations to provide data on aphid migrant numbers without simulating the processes leading to aphid movement [55, 125]. Hendrie *et al.*[31] provided a conceptual framework for the mechanistic simulation of aphid migration using trajectory modelling (there are now examples of this type of model applied to *R. padi*[95, 126]), *R. maidis* and *S. graminum*[13], with the same technique applicable across a wide range of species). Simulation models of the spread of BYDV have tended to be at the field scale, with aphid vector immigration as an input, usually estimated from suction trap data or field survey e.g. [127].

**Table 1:** **Cereal aphid models simulating aphid movement or timing of arrival in crops**

Model characteristics	Aim	Country	Scale	Phase(s) of the transport process included	Reference
Turbulent advection simulation/Lagrangian stochastic	To investigate aerial density profiles in relation to simplified aphid behaviours	UK	Long distance migration	Transport in Atmosphere	[64]
Atmospheric trajectory model of dispersal	To estimate migration pathways	Finland	Long distance migration	Transport in Atmosphere	[95]
Trajectory	Modelling aphid migration from source to sink	Illinois, USA	Long distance migration	Transport in Atmosphere	[13, 31, 106]
Trajectory coupled to cohort-based population dynamics	Mechanistic simulation of aphid population dynamics at source and factors leading to take-off, coupled to wind a trajectory simulation model to estimate potential long distance movement risk from irrigated pastures to crops.	South-western Australia	Long distance migration	Source, Transport in Atmosphere, Initial Distribution	[126]
Large-scale: Diffusion–advection-reaction equations	To simulate the landing rate of *Sitobion avenae* in crop fields across landscapes. Explores landing behaviours and responses to landscape (e.g. wavelengths).	France	Landscape (multi-scale)	Initial Distribution	[123]
Small-scale: cellular automata incorporating behavioural rules.					
Hierarchical Bayesian	Driven by field observations to gain knowledge on processes such as insect landing and mortality	Germany	Within-field	Initial Distribution	[55]. See also [125, 128, 129]
Analytical regression	Prediction of the timing of migration into crops from primary host (holocyclic populations only)	Denmark/Scandinavia	Within-field	Initial Distribution	[130, 131]
Analytical regression	Prediction of the timing of migration into crops from primary host (holocyclic populations only) – requires suction trap data	Sweden	Within-field	Initial Distribution	[132]
Analytical regression	Prediction of the timing of migration into autumn crops – requires suction trap data	Wales	Within-field	Initial Distribution	[92]
Analytical regression	Prediction of the timing of migration into autumn crops – requires suction trap data	UK	Within-field	Initial Distribution	[133]
Analytical regression	Prediction of the timing of migration into spring crops – requires suction trap data	UK	Within-field	Initial Distribution	[134, 135]
Individual-based	Stochastic wind-driven dispersal model to examine difference in dispersal and population dynamics depending on pesticide regime	UK	Small landscape	Local Movement	[76]
Cohort-based population dynamics model (STELLA)	Population dynamics model that simulates immigration from a ‘background’ source population. Spatial variation in immigration at the regional scale driven by differences in soil moisture levels.	South-western Australia	Within-field	Initial Distribution (from local source)	[136]
Analytical mathematical model	Estimation of the percentage of plants infected with BYDV, given the number of aphids per plant. Distinction between alate migrant transmission and apterous transmission.	UK	Within-field	Initial Distribution, Local Movement	[137]
Cohort-based	Aphid population dynamics, local dispersal and virus sub-models.	UK	Within-field/small landscape	Local Movement	[138]. See also [69, 139]
Cellular Automata	Rate of spread of BYDV from an origin cell, based on probabilities of infection transferring to the next cell (combined with field observations).	UK	Within-field	Local Movement	[140]
Individual-based	Simplified model of aphid population dynamics and virus transmission from plant to plant. Focus on computing methods rather than ecology.	UK	Within-field/small scale	Local Movement	[141, 142]
Analytical probabilistic model and Markov chain model of disease transmission. Individual-based aphid movement through field.	Examines aspatially the implications of vector preference for diseased or healthy hosts on the spread of BYDV. A Markov chain model and a stochastic individual-based model examine disease transmission and the effects of spatial patchiness.	USA	Non-spatial (analytical) and spatial within-field (Markov chain).	Local Movement	[143] see also [144]
Artificial Neural Networks and multiple regression	Aphid autumn flight timing/numbers. No BYDV.	New Zealand	Autumn flight	Source	[145]
Analytical linear and probit models	Soybean aphid early season colonisation of fields from overwintering hosts.	Canada	Spring flight. Within-field.	Source, Local Movement	[146]

The simulation models listed in Table 1 span a broad range of techniques, from analytical approaches to highly mechanistic individual-based models. There are pros and cons to the use of each of these methods, as discussed in recent guidelines by Parry *et al.* applicable across the general field of pest modelling, where some approaches may be better applied than others at certain scales, given the modelling objective and constraints [147]. Thus, in order to effectively simulate the full aerial transport process, it may be necessary to take a hybrid modelling approach to ensure the most appropriate techniques are applied (for example a coupled cohort-based or analytical population dynamics model with an atmospheric trajectory model [126]).

There are also models that have been developed for other aphid species that are not pests of cereal crops that are of note. For example, local scale movement of *Aphis gossypii* is simulated within a melon crop field using an individual-based modelling approach to give a sophisticated theoretical account of the evolution of dispersal strategies for this species [148]. Also of note is the use of back-trajectory modelling to relate spring low-level jet (LLJ) streams to intensity of *Myzus persicae* flight activity in the northern USA, which were then used to inform a simple linear regression model to project aphid population growth at crop sites in relation to the cumulative duration of LLJ events [105]. This approach was also applied to cereal aphid species *R. padi*, *R. maidis*, *S. graminum* and *S. avenae*, however no consistent relationship could be found between the duration of LLJ events and aphid population growth for those species [105], contrary to the findings of Irwin and Thresh [13].

Overall, although simulation models developed to date span the full range of the atmospheric transportation process, they tend to focus on each individual component, rather than the process as a whole. However, along with the depth of knowledge available on the mechanisms of aphid flight, this modelling toolbox now gives the opportunity to move towards simulation models that are capable of integrating the mechanisms of flight initiation, the transportation process and the arrival of aphids on hosts, to better inform area-wide pest management strategies.

## Conclusions

There is an extremely rich literature spanning decades that is relevant to understanding many aspects of cereal aphid flight and migration. Some aspects of movement have been studied multiple times which increases confidence in our knowledge, such as the threshold wind speed of 8 kmh^-1^ that restricts flight initiation. However, although this threshold has emerged quite clearly from a number of studies (primarily conducted in the laboratory), there is the issue that studies over a longer time period and from the field show that aphids will take flight even in high wind speeds; therefore when flight thresholds have not been put to the test in so many ways we should continue to question them (for example humidity).

Such a wealth of information can be overwhelming, particularly to construct a simulation model of aphid flight. To this end, I distil cereal aphid migration into four phases, and conceptualize the flight of cereal aphids as following four key principles, around which are ‘nuances’ of aphid flight behaviour that might be incorporated into a model (but about which there is greater uncertainty).

The overall conceptualization of aphid flight has changed over the years, from assuming that migration was common to now considering that it is the exception, rather than the rule, predominantly occurring in newly emerged alate adults. Furthermore, there still remain some aspects to aphid flight that we have only recently realised, having made assumptions for many years that are now considered incorrect. For example, there is now evidence of the ability of aphids to control their elevation in an air-column, questioning the assumption that their flight behaviour during the transport phase can be assumed to be completely ‘passive’ [64].

Despite such rich information in the literature, there are still significant gaps in our knowledge. An important gap seems to be quantification of humidity and high temperature thresholds for flight initiation: likely to become increasingly significant under global climate change for regions that are already pushing the climatic niche for some aphid species, such as *R. padi* in Australia [149]. Landing cues and the processes that control the termination of flight are an obvious gap where knowledge is poor and empirical studies are difficult to conduct, but observational data is available. The multi-scale model by Ciss *et al*. [123] explores multiple hypotheses about landing cues and demonstrates the potential of simulation modelling to advance our understanding of this process, particularly if tested against observational data. It is important to build on this with further simulation modelling studies coupled with observational or empirical research, as together they provide a powerful tool to test hypotheses about migration patterns observed in space and time.

An important aspect to the integration of models with data is the ability of models to ‘scale-up’ data collected over short time periods or limited spatial scales. Models are increasingly used to explore multiple hypotheses and scenarios, against which we can collect data to verify, potentially using a ‘pattern oriented’ approach to explore the most probable explanations offered by the model in comparison with data [150]. We cannot hope to ever collect enough observational data or conduct empirical studies alone that can give us an understanding of the entire process of cereal aphid migration and flight, however when combined with mechanistic, scalable simulation modelling approaches this becomes achievable. This would lead to an understanding that can help us determine and manage aphid problems not just within-field but at the landscape scale, taking into account source areas to develop management strategies operating at multiple spatial and temporal scales.

Data on various aspects of cereal aphid flight and migration is increasingly available and the range of methodologies with which we can obtain such data has increased in recent times, for example with genetic tools and radar observations. It is important that long-term data collections continue, such as suction trapping data which has been shown to be valuable in regions such as the USA and Europe but is yet to be established in other regions, such as Australia [151].

Finally, the rapid population increase and high mobility of cereal aphid pests are key factors that make them highly damaging; aphids, being so well studied and modelled, can provide a blueprint for identifying the research needs to manage other highly mobile insect pests, that are likely to be less well studied.

## Endnotes

^a^“The trajectory or path of an air parcel is a curve denoting successive three-dimensional positions in time of the air parcel” [152] pp 2.

## Author’s information

The author has worked for CSIRO for the past four years, including a two year CRC for National Plant Biosecurity post-doctoral research position, working on an aphid population dynamics and dispersal model to explore the effects of climate change on plant biosecurity. She completed her Doctorate at the University of Leeds, UK, in late 2006, entitled ‘Effects of Land Management upon Species Population Dynamics: A Spatially Explicit, Individual-based Model’, which focused on the cereal aphid *Rhopalosiphum padi*. Dr Parry graduated from the University of Cambridge, UK, in 2002, achieving an upper-second class Bachelor of Arts with Honours in Geography. She was awarded first-class for her undergraduate dissertation: Modelling the distribution of *Quercus infectoria*, a species of Oak tree, in Cyprus. http://www.csiro.au/people/Hazel.Parry.

## Electronic supplementary material

Additional file 1: **Online supplement.** Quantitative variables useful for the development of mechanistic simulation models of aphid flight, primarily relating to the four general principles given in the paper and should be read in relation to the discussion therein. (DOCX 32 KB)
