# Supplementary material for: Cereal aphid movement: general principles and simulation modelling
Source: Mov Ecol. 2013 Dec 23;1(1):14. doi: 10.1186/2051-3933-1-14 (PMC4337770; doi:10.1186/2051-3933-1-14)
Supplement: Supplementary file 1 — Additional file 1: Online supplement. Quantitative variables useful for the development of mechanistic simulation models of aphid flight, primarily relating to the four general principles given in the paper and should be read in relation to the discussion therein. (DOCX 32 KB) [file 40462_2013_13_MOESM1_ESM.docx]

**ONLINE SUPPLEMENT**

**Quantitative variables useful for the development of mechanistic simulation models of aphid flight, primarily relating to the four general principles given in the paper and should be read in relation to the discussion therein.**

**Table S1: Flight parameters for uplift at the source (flight initiation).** This table summarises key boundary values obtained from the literature for each environmental factor known to affect aphid flight initiation.

| **Parameter** | **Value/Notes** | **Source** |
| --- | --- | --- |
| **Boundary layer** | Above 1m from the ground aphid movement is controlled by the wind | [1] Broadbent (1948); [2] Campbell  and Ridout (2001); [3] Compton (2002); [4] Taylor (1974) |
| **Wind effects** | Aphids can’t fly into wind stronger than approximately 0.5ms^−1^ (2kmh^−1^). | [5] Haine (1955); [6] Loxdale et al. (1993) |
|  | A large number of papers state that aphids do not take off in wind speeds above 8kmh^−1^. | [7] Bottenberg and Irwin (1991); [8] Davies (1939); [5] Haine (1955); [9] Johnson (1962); [10] Kennedy (1990); [11] Kennedy and Booth (1963); [12] Stapley (1949); [13] Thomas and Vevai (1940) |
|  | Evidence from suction traps shows that aphids will fly in high winds. | [14] Johnson (1953) |
|  | High winds delay but do not inhibit take-off. Continuous wind velocities of 5kmh^−1^ caused a delay of 4-10 hrs, and wind speeds of 8 kmh^−1^ caused a delay of 24 hours or more. However, after these extensions migratory flight occurred with regularity, when in a highly active state.  Aphids will still take off in wind velocities of approximately 10-11 kmh^-1^ | [15] Walters and Dixon (1984)  [5] Haine (1955) |
| **Ascent rate** | Migrant alate aphids ascend rapidly up into the atmosphere if they are carried by convective updrafts with rates of ascent measured at up to 3ms^-1^ | [16] Gatehouse (1997) |
|  | Without an updraft, aphids may climb rapidly when in a migratory state, e.g. 0.25ms^-1^ (*Aphis fabae*) | [17] David (1988) |
| **Minimum temperature threshold** | In the UK the temperature threshold for flight varies with season for *R. Padi*:  SPRING: min temp = 14°C; 50% takeoff = 16-17°C; 100% takeoff = 13-14°C  SUMMER: min temp =  11°C; 50% takeoff = 13-14°C; 100% takeoff = 15°C  AUTUMN: min temp  = 3°C; 50% takeoff = 9-10°C; 100% takeoff = 13°C.  Equations given for range of temperatures. | [15] Walters and Dixon  (1984); |
|  | A study in Uppsala, Sweden, showed that the temperature threshold for flight was highest in spring (16-17 °C) when leaving the primary host and lowest in autumn (9-10°C) while the threshold in summer is 13-14°C (flight between grasses). | [18] Wiktelius (1981) |
|  | This may relate to temperatures over a time period (days) | [19] Kleuken, 2009 |
|  | Cereal aphids take off at 16°C  *Aphis fabae* or *Myzus persicae* take off at about 17°C | [20] Klingauf, 1987  [9] Johnson, 1962 |
| **Maximum temperature threshold** | The maximum is presumed to be about 31°C | [21] Robert, 1987;  [15] Walters and Dixon, 1984; [22] De Barro and Maelzer, 1993 (see also references in [19] Kleuken 2009) |
| **Humidity** | Early research showed that humidity should be lower than 70% for migration to occur. | [12] Stapley, 1949; [8] Davies, 1939; [13] Thomas and Vevai, 1940 (all cited by [23] Johnson, 1954) |
|  | After acclimitization aphids will take off readily even if humidity is 50-100% RH, therefore the effect of humidity is considered irrelevant. | [24] Lewis and Siddorn, 1972; |
| **Flight window** | The period between moulting and taking flight varies from 6-36 hours. | Johnson, 1953 |
|  | Wing muscle autolysis occurs in as little as 2-3 days after moult. | [6] Loxdale et al., 1993; [25] Dixon, 1988; [26] Broadbent, 1949; [14] Johnson, 1953; [27] Hardie et al., 1990; [28] Johnson et al., 1957; [29] Cockbain, 1961 |
| **Light** | In general, take-off occurred at light intensities of 1000 lux (approx 3.85 Wm^-2^) and higher, with no upper limit. Laboratory studies have shown lower limits but such limits are considered negligible in the field. | [21] Robert, 1987 |

**Table S2: Flight parameters for atmospheric transportation.** This table summarises key boundary values obtained from the literature for each environmental factor known to affect aphid atmospheric transportation.

| **Parameter** | **Value** | **Source** |
| --- | --- | --- |
| **Flight speed** | Maximum = 0.9 ms^-1^ (3.24 kmh^-1^)  Flight speed ranges from 0.8-3.3kmh^-1^ | [3] Compton, 2002  [21] Robert, 1987 |
| **Flight distance** | Most migration is of the order of 20-50km | [30] Ward et al., 1998; |
| **Flight duration** | Average flight time spring migrants *Aphis fabae*: 19.3 min, *R. padi*: 36 min  Average flight time autumn migrants (*Aphis fabae*): 184 min, *R. padi*: 110 min | [31] Nottingham and Hardie, 1989; [32] Nottingham et al., 1991 |
|  | Average flight time is 32-260 mins for flights >1 min duration (average 105 min), *Aphis fabae* | [11] Kennedy and Booth, 1963 |
|  | Estimated percentages for aphids predicted to remain airborne after x seconds: e.g. 0.1% aphids remain airborne after 3 hours. | Reynolds and Reynolds, 2009 |
| **Altitude** | Aphids may be carried long distances by winds at 300-1500m | Taylor, 1965: quoted in [24] Lewis and Siddorn, 1972, pp253 |
|  | Insects were common in layers below 1000m, and were observed up to height of about 2.5km | [33] Leskinen, 2011 |
|  | Most winged aphid migrations occur between 0.12 and 1200m of height, in air layers where aphids are randomly distributed by turbulent atmospheric diffusion over a short distance.  Aphid density generally decreases systematically with altitude. | [34] Fabre *et al*. 2010  [35] Isard *et al.* 1990 |
| **Long distance flight termination** | Renewed visual responsiveness to plant-related wavelengths occurs, especially to yellow. Aphids are attracted to wavelengths > 500mμ, especially yellow, also green and orange. Actively bypass blue to ultra violet spectrum. Tested in the field, but some species are less sensitive. | [3] Compton (2002); [36] Kennedy et al. (1961); [6] Loxdale et al. (1993); |

**Table S3: Flight parameters for appetitive flight.** This table summarises key boundary values obtained from the literature for each environmental factor known to affect aphid appetitive movement.

| **Parameter** | **Value** | **Source** |
| --- | --- | --- |
| **Wind effects** | Alate aphids lose control of their flight at wind speeds of around 2kmh^−1^ therefore lower wind speed is required for appetitive flight. | [5] Haine (1955); [6] Loxdale et al. (1993) |
| **Flight speed** | Maximum = 0.9 ms^-1^ (3.24kmh^-1^)  Flight speed ranges from 0.8-3.3kmh^-1^ | [3] Compton, 2002  [21] Robert, 1987 |
| **Flight duration and distance** | To obtain the maximum distance flown by foraging aphids, the maximum flight speed can be multiplied by the total foraging flight time of an aphid, which is about 30-240 minutes (influenced by many factors, e.g. habitat and exhaustion). The resultant maximum distance would be around 200 m (without wind assistance). The majority of flights will be much shorter, just a few metres with flights lasting only a few seconds. | [11] Kennedy and Booth, 1963 |
|  | Apterous aphids walk very small distances from plant to plant (at a speed of around 5-20cm/min), or may ‘run’ from 15-35cm/min. | [37] Hodgson, 1991;  Other references given in [21] Robert, 1987, p299 |
|  |  |  |

**References:**

1. Broadbent L: **Aphis migration and the efficiency of the trapping method.** *Annals of Applied Biology* 1948, **35:**379-394.

2. Campbell CAM, Ridout MS: **Effects of plant spacing an interplanting with oilseed rape on colonisation of dwarf hops by the damson-hop aphid, Phorodon humuli.** *Entomologia Experimentalis et Applicata* 2001, **99:**211-216.

3. Compton S: **Sailing with the wind: dispersal by small flying insects.** In *Dispersal Ecology: the 42nd symposium of the British Ecological Society held at the University of Reading, 2-5 April 2001*. Edited by Bullock JM, Kenward RE, Hails RS. Blackwell Science, Oxford; 2002: 113-133.

4. Taylor LR: **Insect migration, flight periodicity and the boundary layer.** *Journal of Animal Ecology* 1974, **43:**225-238.

5. Haine E: **Aphid take-off in controlled wind speeds.** *Nature* 1955, **175:**474-475.

6. Loxdale HD, Hardie J, Halbert S, Foottit R, Kidd NAC, Carter CI: **The relative importance of short- and long-range movement of flying aphids.** *Biological Review* 1993, **68:**291-311.

7. Bottenberg H, Irwin ME: **Influence of wind speed on residence time of *Uroleucon ambrosiae* alatae (Homoptera: Aphididae) on bean plants in bean moncultures and bean-maize mixtures.** *Environmental Entomology* 1991, **20:**1375-1380.

8. Davies WM: **Studies on aphides infesting the potato crop VII. Report on a survey of the aphis population of potatoes in selected districts of Scotland (25 July-6 August 1936).** *Annals of Applied Biology* 1939, **26:**116-134.

9. Johnson CG: **Aphid Migration.** *New Scientist* 1962, **305:**622-625.

10. Kennedy JS: **Behavioural post-inhibitory rebound in aphids taking flight after exposure to wind.** *Animal Behaviour* 1990, **39:**1078-1088.

11. Kennedy JS, Booth CO: **Free flight of aphids in the laboratory.** *Journal of Experimental Biology* 1963, **40:**67-85.

12. Stapley JH: *Pests of Farm Crops.* London; 1949.

13. Thomas I, Vevai EJ: **Aphis migration. An analysis of the results of five seasons' trapping in North Wales.** *Annals of Applied Biology* 1940, **27:**393-405.

14. Johnson B: **Flight muscle autolysis and reproduction in aphids.** *Nature* 1953, **172:**813.

15. Walters KFA, Dixon AFG: **The effect of temperature and wind on the flight activity of cereal aphids.** *Annals of Applied Biology* 1984, **104:**17-26.

16. Gatehouse AG: **Behaviour and ecological genetics of wind-borne migration by insects.** *Annual Review of Entomology* 1997, **42:**475-502.

17. David CT, Hardie JIM: **The visual responses of free-flying summer and autumn forms of the black bean aphid, Aphis fabae, in an automated flight chamber.** *Physiological Entomology* 1988, **13:**277-284.

18. Wiktelius S: **Diurnal flight periodicities and temperature thresholds for flight for different migrant forms of *Rhopalosiphum padi* L. (Hom., Aphididae).** *Zeitschrift für Angewandte Entomologie* 1981, **92:**449-457.

19. Kleuken AM, Hau B, Freier B, Friesland H, Kleinhenz B, Poehling H-M: **Comparison and validation of population models for cereal aphids.** *Journal of Palnt Diseases and Protection* 2009, **116:**129-140.

20. Klingauf A: **Host Plant Finding and Acceptance.** In *Aphids: Their Biology, Natural Enemies and Control.* Edited by Minks AK, Harrewijn P: Elsevier, Amsterdam; 1987: 209-223: *World Crop Pests*].

21. Robert Y: **Aphids and their Environment: Dispersion and Migration.** In *Aphids: Their biology, natural enemies and control.* Edited by Minks AK, Harrewijn P: Elsevier, Amsterdam; 1987: 299-313: *World Crop Pests*].

22. De Barro P, Maelzer DA: **Influence of high temperatures on the survival of *Rhopalosiphum padi* (L.)(Hemiptera: Aphididae) in irrigated perennial grass pastures in South Australia.** *Australian Journal of Zoology* 1993, **41:**123-132.

23. Johnson CG: **Aphid Migration in Relation to Weather.** *Biological Reviews* 1954, **29:**87-118.

24. Lewis T, Siddorn JW: **Measurement of the physical environment.** In *Aphid Technology.* Edited by van Emden HF. London: Academic Press; 1972: 235-273

25. Dixon AFG: *Aphid Ecology.* 2nd Edition edn. London: Chapman and Hall; 1988.

26. Broadbent L: **Factors Affecting the Activity of Alatae of the Aphids *Myzus-Persicae* (Sulzer) and *Brevicoryne-Brassicae* (L).** *Annals of Applied Biology* 1949, **36:**40-62.

27. Hardie J, Mallory ACL, Quashie-Williams CA: **Juvenile hormone and host-plant colonization by the black bean aphid, *Aphis fabae.*** *Physiological Entomology* 1990, **15:**331-336.

28. Johnson B: **Studies on the degeneration of the flight muscles of alate aphids. I. A comparative study of the occurrence of muscle breakdown in relation to reproduction in several species.** *Journal of Insect Physiology* 1957, **1:**248-256.

29. Cockbain AJ: **Viability and fecundity of alienicolae of *Aphis fabae* Scop. after flights to exhaustion.** *Journal of Experimental Biology* 1961, **38:**181-187.

30. Ward SA, Leather SR, Pickup J, Harrington R: **Mortality during dispersal and the cost of host specificity in parasites: how many aphids find hosts?** *Journal of Animal Ecology* 1998, **67:**763-773.

31. Nottingham SF, Hardie J: **Migration and targeted flight in seasonal forms of the black bean aphid, *Aphis fabae*.** *Physiological Entomology* 1989, **14:**451-458.

32. Nottingham SF, Hardie J, Tatchell GM: **Flight behaviour of the bird cherry aphid, *Rhopalosiphum padi*. .** *Physiological Entomology* 1991, **16:**223–229.

33. Leskinen M, Markkula I, Kostinen J, Pylkko P, Ooperi S, Siljamo P, Ojanen H, Raiskio S, Tiilikkala K: **Pest insect immigration warning by an atmospheric dispersion model, weather radars and traps.** *Journal of Applied Entomology* 2011, **135:**55-67.

34. Fabre F, Dedryver CA, Plantegenest M, Hullé M, Rivot E: **Hierarchical Bayesian Modelling of plant colonisation by winged aphids: Inferring dispersal processes by linking aerial and field count data.** *Ecological Modelling* 2010, **221:**1770-1778.

35. Isard SA, Irwin ME, Hollinger SE: **Vertical distribution of aphids (Homoptera: Aphididae) in the planetary boundary layer.** *Environmental Entomology* 1990, **19:**1473–1484.

36. Kennedy JS, Booth CO, Kershaw WJS: **Host finding by aphids in the field III. Visual attraction.** *Annals of Applied Biology* 1961, **49:**1-21.

37. Hodgson C: **Dispersal of apterous aphids (Homoptera: Aphididae) from their host plant and its significance.** *Bulletin of Entomological Research* 1991, **81:**417-427.
